# TLR2, TLR4 and the MyD88 Signaling Are Crucial For the *In Vivo* Generation and the Longevity of Long-Lived Antibody-Secreting Cells

**DOI:** 10.1371/journal.pone.0071185

**Published:** 2013-08-05

**Authors:** Evilin Naname Komegae, Lidiane Zito Grund, Monica Lopes-Ferreira, Carla Lima

**Affiliations:** Immunoregulation Unit, Special Laboratory of Applied Toxinology, Butantan Institute and Department of Immunology, University of São Paulo, São Paulo, Brazil; Institut National de la Santé et de la Recherche Médicale, France

## Abstract

This study was undertaken to gain better insights into the role of TLRs and MyD88 in the development and differentiation of memory B cells, especially of ASC, during the Th2 polarized memory response induced by Natterins. Our *in vivo* findings demonstrated that the anaphylactic IgG1 production is dependent on TLR2 and MyD88 signaling, and that TLR4 acts as adjuvant accelerating the synthesis of high affinity-IgE. Also, TLR4 (MyD88-independent) modulated the migration of innate-like B cells (B1a and B2) out of the peritoneal cavity, and the emigration from the spleen of B1b and B2 cells. TLR4 (MyD88-independent) modulated the emigration from the spleen of Bmem as well as ASC B220^pos^. TLR2 triggered to the egress from the peritoneum of Bmem (MyD88-dependent) and ASC B220^pos^ (MyD88-independent). We showed that TLR4 regulates the degree of expansion of Bmem in the peritoneum (MyD88-dependent) and in BM (MyD88-independent) as well as of ASC B220^neg^ in the spleen (MyD88-independent). TLR2 regulated the intensity of the expansion of Bmem (MyD88-independent) and ASC B220^pos^ (MyD88-dependent) in BM. Finally, TLR4 signals sustained the longevity of ASC B220^pos^ (MyD88-independent) and ASC B220^neg^ into the peritoneum (MyD88-dependent) and TLR2 MyD88-dependent signaling supported the persistence of B2 cells in BM, Bmem in the spleen and ASC B220^neg^ in peritoneum and BM. Terminally differentiated ASC B220^neg^ required the cooperation of both signals through TLR2/TLR4 via MyD88 for longevity in peritoneum, whereas Bmem required only TLR2/MyD88 to stay in spleen, and ASC B220^pos^ rested in peritoneum dependent on TLR4 signaling. Our data sustain that earlier events on memory B cells differentiation induced in secondary immune response against Natterins, after secondary lymph organs influx and egress, may be the key to determining peripheral localization of innate-like B cells and memory B cells as ASC B220^pos^ and ASC B220^neg^.

## Introduction

Immunological memory is a key hallmark of adaptive immune responses. Maintenance of high serum antibodies (Abs) level by long-term is imperative for improving vaccine development, but uncontrolled generation of autoantibodies results in autoimmune diseases. Interestingly, the majority of allergen-specific IgE in the blood of allergic patients [1], as well as the production of anti-RNA and anti-cardiolipin Abs in systemic lupus erythematosus patients [2] are produced by long-lived antibody-secreting cells (ASC CD138^pos^) found in both secondary lymphoid organs and bone marrow (BM).

Protective memory is mediated by ASC that are terminally differentiated and continue secrete Abs in specific microenvironment. The loss of expression of B220 molecule and the gain of expression of others molecule as CD138, CD43, CD38, CD62L and CD93 characterize ASC. Also, the reactive memory is mediated by memory B cells (Bmem) that proliferate and differentiate into ASC upon exposure to antigens [3], [4]. Bmem express high affinity surface immunoglobulin (Ig), CD80, CD86, CD95, CD19, B220, CD27 (human) and high levels of intracellular transcription factor PAX5 [5], [6]. Both type of memory cells can be generated from innate-like B cells as B1 and conventional B (B2) [7].

For non-proliferating ASC, maintenance would completely depend on cell survival that is conferred by combined cell intrinsic and extrinsic factors. The intrinsic genetic program (Blimp-1, Bach2, Bcl-6, IRF4, Xbp1, and Pax5,) that drives the differentiation of ASC is becoming clear [8]. Less clear are the modes of action of extrinsic signals, as well as their associated downstream signaling pathways, in initiating or enhancing this important transition. A strong signal through the antigen-specific B cell receptor (BCR) is thought to signal Bcl-6 degradation and, thus, de-repression of B lymphocyte-induced maturation protein 1 - Blimp-1 [9]. Bacterial products such as LPS can drive T-independent ASC differentiation, whereas CD40L and T cell–derived cytokines signal T-dependent ASC differentiation, particularly IL-4, IL-5, and IL-21 in the mouse and IL-6 and IL-10 in humans.

Recently, we have provided evidence in BALB/c mice that IL-17A as well as IL-5 produced in a context of chronic inflammatory response against venom proteins of *Thalassophryne nattereri* (V*Tn*) Brazilian fish directly influence the production of specific IgE Abs and the differentiation and maintenance of ASC with B220^neg^ phenotype in inflamed peritoneal cavity [10], [11]. Using Natterins, a class of toxins with proteolytic activity isolated in the V*Tn* [12], [13], we also demonstrated in BALB/c mice that the proteolytic activity of Natterins besides inducing a Th2 response with plasmatic titers of high affinity antigen-specific IgE over extended periods is sufficient for the generation of survival signals that contribute to the formation of a molecular survival niche in secondary lymphoid organ, essential for the long-term maintenance of terminally differentiated ASC (*personal communication*). Therefore, the use of venom and toxins isolated from V*Tn* provides an interesting scenario for studying the signals involved in the differentiation and survival of the memory B cell compartment.

A striking characteristic of B cells is the expression of a clonally rearranged BCR in conjunction with the expression of one or more members of Toll-like receptors (TLRs). TLRs represent a family of evolutionary conserved pattern recognition receptor (PRR) that recognizes a wide range of microbial ligands [14], [15] and use Toll–IL-1 receptor (TIR) domain-containing adapters, such as myeloid differentiation primary response protein 88 (MyD88) and TIR domain-containing adapter inducing IFN-β (TRIF), to induce activation of transcription factors, including NF-κB, MAP kinases, and IFN regulatory factors [16]. This dual expression pattern permits B cells to uniquely integrate both antigen-specific signals and danger signals via these key receptor systems.

Recognition of pathogen-associated molecules by TLRs expressed on “classic” innate cells, such as dendritic cells (DC) and macrophages, triggers their maturation leading to initiation of antigen-specific adaptive immune responses through T cell activation. Furthermore, direct signals through TLRs expressed on B cells play an important role in the activation and optimal Abs production to T-dependent antigens [17]. In B cells, TLRs activation results in the up-regulation of activation markers, proliferation, cytokine secretion, terminal differentiation and Blimp-1 expression and, finally, Ig secretion [18]–[23].

The importance of the integration of signaling pathways downstream of BCRs and TLRs in modulating an optimal Abs production to T-dependent antigens by the expansion of the memory B cell compartment, especially the generation and maintenance of ASC have been insufficiently recognized. This study was undertaken to gain better insights into the role of TLRs and MyD88 in the development and differentiation of memory B cells, especially of ASC, during the Th2 polarized memory immune response induced by fish proteases Natterins.

## Materials and Methods

### Mice

Male C3H/HePas (TLR4 wild type - *WT*), C3H/HeJ (TLR4 mutant), C57BL/6 (*WT*), C57BL/6 TLR2 knockout (*KO*) and C57BL/6 (MyD88 *KO*) mice (5–6 weeks old) were obtained from a colony at Institute of Biomedical Sciences II, University of São Paulo, São Paulo, Brazil. Mice were housed in a laminar flow holding unit (Gelman Sciences, Sydney, Australia) in autoclaved cages on autoclaved bedding, in an air-conditioned room in a 12-hour light/dark cycle. Irradiated food and acidified water were provided *ad libitum*. This study was carried out in strict accordance with the recommendations in the Guide for the Care and Use of Laboratory Animals of the Brazilian College of Animal Experimentation. The protocol was approved by the Committee on the Ethics of Animal Experiments of the Butantan Institute (Permit Number: 504/08) and of University of São Paulo (Permit Number: 747/10). All surgery was performed under sodium pentobarbital anesthesia, and all efforts were made to minimize suffering.

### Natterins Preparation

*T. nattereri* fish venom was obtained from fresh captured specimens at the Mundau Lake in Alagoas, state of Brazil with a trawl net from the muddy bottom of lake. No protected specimens were captured and fish were transported to Immunoregulation Unit of Butantan Institute. All necessary permits were obtained for the described field Studies (capture, conservation and venom collection - IBAMA Permit Number: 16221-1). Venom was immediately extracted from the openings at the tip of the spines by applying pressure at their bases. After centrifugation, venom was pooled and stored at −80°C before use according to Lopes-Ferreira et al., [24]. After that fish were anesthetized with 2-phenoxyethanol prior to sacrifice by decapitation. The venom was fractionated by cation exchange chromatography, using the fast protein liquid chromatography system (FPLC - Pharmacia, Uppsala, Sweden). Immediately before chromatography, 2 mg venom was diluted in 500 µL of buffer A (20 mM Tris-hydroxymethylaminomethane, pH 8.3) and the solution centrifuged at 10.000 g for 5 minutes. The sample was applied on Mono S column HR 5/5 equilibrated with buffer A. The retained proteins are eluted with a linear gradient of NaCl (0–2 M) and collected at a flow rate of 1.0 mL/min. The elution profile was determined by measuring absorbance at 280 nm. Fractions 1–4, except the fifth, corresponding to Natterins were pooled, dialyzed against 50 mM Tris/HCl pH 7.4 and evaluated with respect to its protein content and kept at −20°C until use. The Natterins obtained were analyzed by polyacrylamide gel electrophoresis with 12% SDS (SDS-PAGE) according to Laemmli [25].

Endotoxin content was evaluated (resulting in a total dose <0.8 pg/mL LPS) with QCL-1000 chromogenic *Limulus amoebocyte* lysate assay (Bio-Whittaker) according to the manufacturer's instructions. The total amount of protease was determined using quenched fluorescein-kininogen substrate (Molecular Probes). Enzymatic release of fluorescent signals was quantified by a microplate fluorometer BMG Fluostar Galaxy V4.30.0 (BMG Labtechnologies) according to the manufacturer's instructions and data was expressed as percentage of inhibition [24].

### Induction of Memory Immune Response by Natterins

Groups of 5 mice were immunized with intraperitoneal (i.p.) injections of 10 µg of Natterins on day 0 and boosted on days 7, 21 and 28. The first immunization was given in 1.6 mg of aluminum hydroxide (Al(OH)_3_) as adjuvant. Mice injected only with adjuvant were considered as the control group. Blood samples were collected at 48, 74, and 120 days after the first immunization and stored at −20°C until analysis. Mice were killed by the injection of lethal dose of sodium pentobarbital anesthesia, and peritoneal fluid, spleen and BM were harvested at various time points, and single cell suspensions were prepared as described previously [26].

### FTY-720 Treatment of Natterins-immunized BALB/c mice

Groups of 5 BALB/c mice were treated with i.p. injection of 0.2 mL Fingolimod (FTY720 (hydrochloride, CAS 162359-56-0_Cayman Chemical) at 1 mg/Kg according to Cinamon et al. [27], 30 min before Natterins immunization on days 0, 7 and 21. Mice were killed at day 28 by the injection of lethal dose of sodium pentobarbital anesthesia, and peritoneal fluid, spleen and bone marrow were harvested for a single cell suspension preparation.

### Cell Preparation

The cells were obtained at 48, 74 and 120 d after the first immunization of mice. Peritoneal cells were obtained by wash with 5 mL of the RPMI 1640 medium. Spleens were dissociated into single cell suspensions by mechanical disruption in cell strainer (BD Falcon). BM cells were obtained by flushing femurs of the mice. Erythrocytes in spleens and BM were lysed with 0.14 M NH_4_Cl and 17 mM Tris-HCl (pH 7.4). The identification of differential subtypes of innate-like B cells or Bmem and ASC was conducted as described earlier [11].

### Flow Cytometry Analysis

For surface staining single-cell suspensions were treated with 3% mouse serum of naive mice to saturate Fc receptors followed by the staining by fluorescence conjugated Abs: Rat IgG2bk FITC-anti-mouse CD3, Armenian hamster IgG1y2 FITC-anti-mouse CD19, Rat IgG2ak PE-anti-mouse CD5, Rat IgG2ak PE-anti-mouse CD23, Goat IgG2bk PE-anti-mouse IgG [specific for IgG1, IgG2a, IgG2b and IgG3], Rat IgG2bk PE-anti-mouse CD11b, Rat IgG2ak PerCP-Cy5-anti-mouse CD45R/B220, Rat IgG2ak FITC-anti-mouse CD43, Rat IgG2ak PE-anti-mouse CD138, and Armenian hamster IgG APC-anti-mouse CXCR3 and Rat IgG2bk APC-anti-mouse CXCR4 for 30 min on ice. Cells were washed three times in PBS 1% BSA. Negative controls were used to set the flow cytometer photomultiplier tube voltages, and single-color positive controls were used to adjust instrument compensation settings. The cells were examined for viability by flow cytometry using forward/side scatter characteristics or 7-AAD exclusion. Data from stained samples were acquired using a FACSCalibur flow cytometer equipped with CellQuest software (BD Biosciences) and were analyzed using CellQuest Software (Becton-Dickinson, San Jose, CA).

### Intracellular Cytokine Staining

Analysis of cytokine synthesis at the single-cell level was performed by FACSCalibur flow cytometer equipped with CellQuest software (BD Biosciences) and were analyzed using CellQuest Software (Becton-Dickinson, San Jose, CA). Freshly isolated splenic cells at 1×10^7^ cells/mL from Natterins-immunized mice treated or not with FTY720 obtained at day 28 after immunization were restimulated for 16 h at 37°C, 5% CO_2_ with a cell stimulation cocktail containing PMA at 20 ng/mL and ionomycin at 1 µM in the presence of brefeldin A and monesin at 10 µg/mL. Subsequently, after washing and fixation, different subtypes of cells as CD4^pos^ T cells, Ly6C^pos^ macrophages and CD11b^pos^CD11c^pos^ DC were assessed and for intracellular content of IFN-γ, IL-17A, IL-4, IL-13 and IL-10.

### Titration of Total IgE and Specific IgG1 and IgG2a by ELISA

Blood samples were obtained by the puncture of the right ventricle of immunized mice. ELISA for detection of Abs was performed as described [28]. Microtitre 96-well plates (3590 - Costar, Cambridge, MA, USA) were coated with Natterins at 3 µg/mL or BSA (1 µg/mL) (negative control) in PBS for 18 h at 4°C. For blocking, the wells were washed with PBS, and incubated with 200 µL of 10% BSA in PBS for 3 h at 37°C. After washing, plasma were tested for IgG1 or IgG2a Abs using biotinylated goat anti-mouse IgG1 (31236) or IgG2a (31237) antiserum from Thermo Scientific Pierce (Rockford, IL USA) for 1 h at room temperature. The reactions were developed with streptavidin-horseradish peroxidase complex (Sigma), O-phenylenediamine (OPD) and H_2_O_2_ and the plates were read at 490 nm on an automated ELISA reader (Spectramax, Molecular Devices). The results were expressed as the mean ± SEM absorbance. An IgE-specific ELISA was used to quantitate total-IgE Ab levels in plasma using matched Ab pairs (553413 Pharmingen and 1130-08 Southern Biotech.), according to the manufacturer's instructions. Samples were quantified by comparison with a standard curve of IgE (0313ID, Pharmingen).

### Passive Cutaneous Anaphylaxis (PCA) for Specific Antibodies

The anaphylactic activity of IgG1 was evaluated by PCA reactions in mice as described by Ovary [29]. Mice were previously shaved and injected intradermically (50 µL) with three serial dilutions of plasma (inactivated for 1 h at 56°C) in each side of the dorsal skin. For IgE titration, PCA reactions were performed in rats using non-inactivated plasma, according to Mota & Wong [30]. After 2 or 18 h they were challenged i.v. with 50 µg of Natterins +0.25% of Evans blue solution. All tests were made in triplicate and PCA titers were expressed as the reciprocal of the highest dilution that gave a lesion of >5 mm in diameter. The detection threshold of the technique was established at 1∶5 dilutions.

### Statistical Analysis

All values were expressed as mean ± SEM. Experiments were performed three times. Parametric data were evaluated using analysis of variance, followed by the Bonferroni test for multiple comparisons. Non-parametric data were assessed using the Mann-Whitney test. Differences were considered statistically significant at *p*<0.05. The SPSS statistical package (Release 13.0, Evaluation Version, 2004) was employed.

## Results

### TLR2 and MyD88 positively regulated the production of anaphylactic-IgG1 induced by Natterins, and the production of anaphylactic-IgE is accelerated by TLR4

Differentiation of antigen-specific B cells, including Ig class switch DNA recombination (CSR) and somatic hypermutation (SHM), is critical for the immune response and depends on the integration of signals from the BCR, CD40, TLRs and cytokine receptors. Previously, we showed in BALB/c mice that the development of IgE-secreting B cells Th2-dependent is strongly driven by proteolytic activity of the Natterins. To investigate the role of TLR4, TLR2 and the main adaptor molecule involved with TLRs signaling (MyD88) in the generation of humoral response to Natterins functionally active, we immunized *WT*, mutant, and *KO* mice with Natterins. The persistence of plasmatic titers of specific-Abs mirrored the GC reactions, and then ELISA or PCA were used for measurement of the specific reactivity for each Abs isotype in sera from groups of mice (*n* = 5).

**Figure 1:**
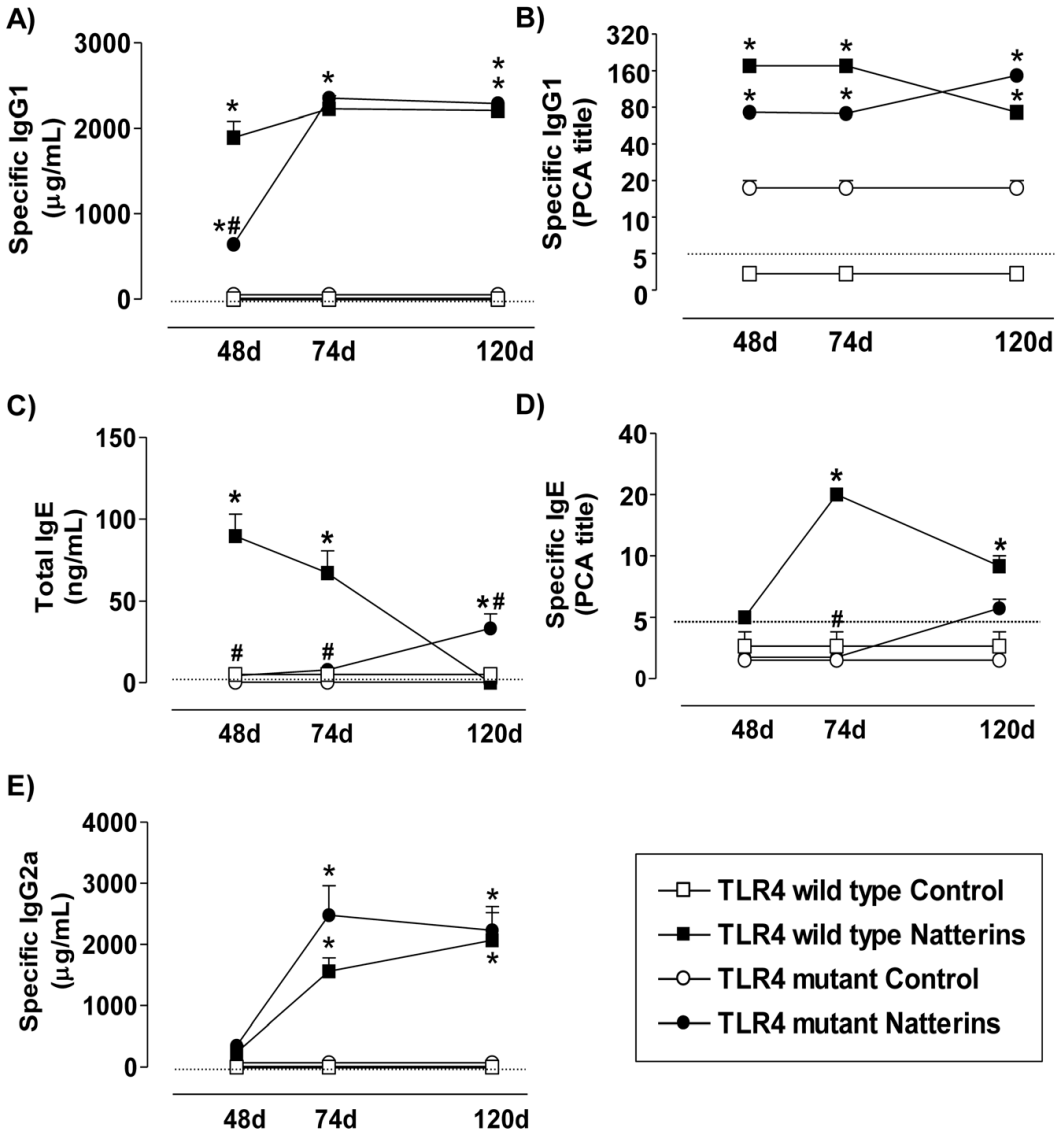
The production of anaphylactic-IgE is accelerated by TLR4. TLR4 *WT* (C3H/HePas) and TLR4 deficient (C3H/Hej) mice were immunized with Natterins on day 0 and boosted 7, 21 and 28 days later. Mice were bled at days 48, 74 and 120 to analyze Natterins specific-IgG1 and IgG2a and total-IgE Abs titers by ELISA. The anaphylactic IgG1 and IgE Abs were examined by PCA. Each group consisted of at least five male mice, and representative data from three repeated experiments are shown. *p<0.05 compared to control mice; and ^#^ *p*<0.05 compared to *WT* mice immunized with the Natterins. The dotted line represents the lower limit of detection methods.

**Figure 2:**
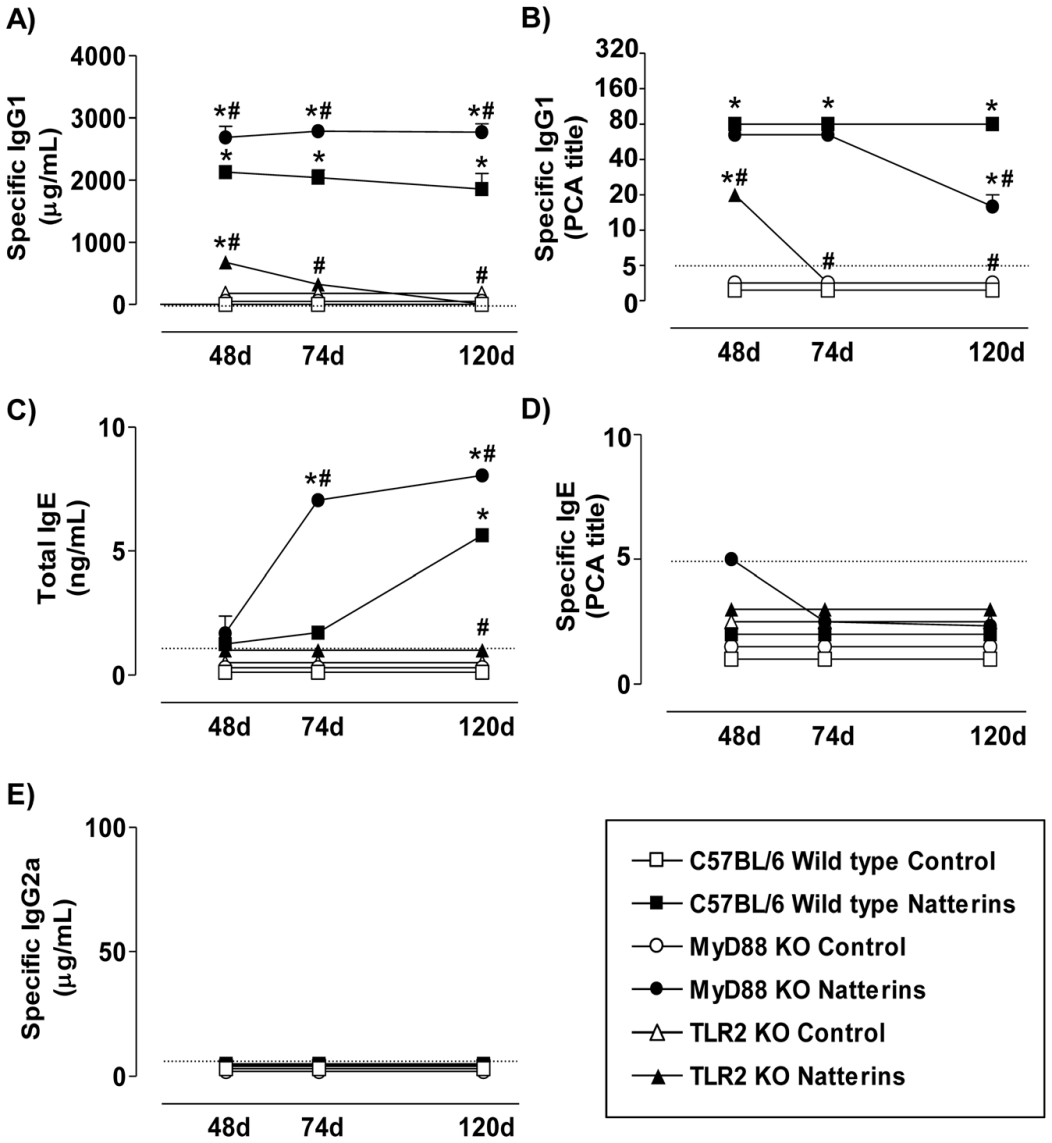
TLR2 and MyD88 positively regulate the production of anaphylactic-IgG1 induced by Natterins. C57BL/6 *WT*, TLR2 *KO* and MyD88 *KO* mice were immunized with Natterins on day 0 and boosted 7, 21 and 28 days later. Mice were bled at days 48, 74 and 120 to analyze Natterins specific-IgG1 and IgG2a and total IgE Abs titers by ELISA. The anaphylactic IgG1 and IgE Abs were examined by PCA. Each group consisted of at least five male mice, and representative data from three repeated experiments are shown. *p<0.05 compared to control mice; and ^#^ *p*<0.05 compared to *WT* mice immunized with the Natterins. The dotted line represents the lower limit of detection methods.

TLR4 *WT* and TLR4 mutant of C3H strain mice (except by 48 d) produced a robust and persistent specific-IgG1 response to Natterins until 120 d compared to control mice (Fig. **1** ***A***). Also, both group of immunized-mice presented high and similar titers of anaphylactic-IgG1 until 120 d determined by PCA (Fig. **1** ***B***). In contrast, TLR2 deficiency in C57BL/6 strain resulted in a drastic reduction of both specific- (Fig. **2** ***A***) and anaphylactic-IgG1 Abs (Fig. **2** ***B***) compared with *WT* immunized-mice. The deficiency of MyD88 in C57BL/6 mice immunized with Natterins resulted in an increase in the levels of specific-IgG1 (Fig. **2** ***A***) and in a decrease in the late production of anaphylactic-IgG1 Abs at 120 d (Fig. **2** ***B***) compared with *WT* immunized-mice.

The production of moderated levels of total-IgE until 74 d in TLR4 *WT* immunized with Natterins was abolished in TLR4 mutant immunized-mice, which initiated the production of this Abs at 120 d (Fig. **1** ***C***). The production of low levels of total-IgE in C57BL/6 *WT* exclusively at 120 d after immunization with Natterins was intensified in C57BL/6 MyD88 *KO* immunized-mice (74 and 120 d) and abolished in C57BL/6 TLR2 *KO* during the entire course of response (Fig. **2** ***C***).

The production of moderate titers of anaphylactic-IgE in the later periods of the response in TLR4 *WT* immunized-mice was abolished in TLR4 mutant immunized-mice (Fig. **1** ***D***), and anaphylactic-IgE Abs were not detected in C57BL/6 strain, *WT* or *KO* immunized-mice (Fig. **2** ***D***).

Comparing the results of specific-IgG2a Ab levels of TLR4 *WT* and TLR4 mutant mice, we showed that the absence of functional TLR4 did not influence the synthesis of this type of Ab (Fig. **1** ***E***), and immunization of C57BL/6 *WT* or *KO* mice with Natterins did not induce the production of specific-IgG2a Abs (Fig. **2** ***E***).

These results showed the involvement of TLR2 in the positive control of specific-IgG1, anaphylactic-IgG1 and total-IgE, and also the positive control of TLR4 in the anaphylactic-IgE production induced by Natterins. MyD88 positively regulates the late phase of anaphylactic-IgG1 production. Also, this adaptor molecule is involved in negative control of specific-IgG1 and total-IgE production during entire course of response.

### The transient expansion of B1a cells in spleen and bone marrow is positively mediated by TLR2 and MyD88

The role of B cell subsets in humoral response may depend on their environmental niche and/or the stage of memory response. B1 cells express various TLRs (TLR1, 2, 3, 4, 7, 8, and 9) [31]; and are more prone to terminal ASC differentiation than B2 cells upon TLRs stimulation [32]. B1 cell activation and B1 cell mediated autoantibody production are augmented upon stimulation via TLR4 or TLR9 [33], [34]. Also, TLRs signaling in B1 cells plays an important role in the clearance of various pathogens such as influenza virus, pneumococcus, and *Borrelia* spp [35]–[37].

To further determine whether TLR4, TLR2 and MyD88 signaling are required for B1a cells subtype response to Natterins, we examined the ability of TLR4 mutant, TLR2 *KO*, MyD88 *KO* and *WT* mice to generate changes in absolute number of B1a cells population, by flow cytometry analysis.

B1 cells can be distinguished by differential expression of traditional B cell markers as B220^low^CD19^high^IgM^high^IgD^low^CD23^neg^. B1 cells themselves are comprised of two different populations, B1a and B1b, which are CD5^pos^ and CD5^neg^, respectively. Here B1a cells were quantified as B220^low^CD5^pos^ from a negative-CD3 gated cells in different compartments of C3H or C57BL/6 (*WT* or *KO*) mice as inflamed peritoneal cavity; the secondary lymphoid organ as spleen and in BM, an important survive niche.

**Figure 3:**
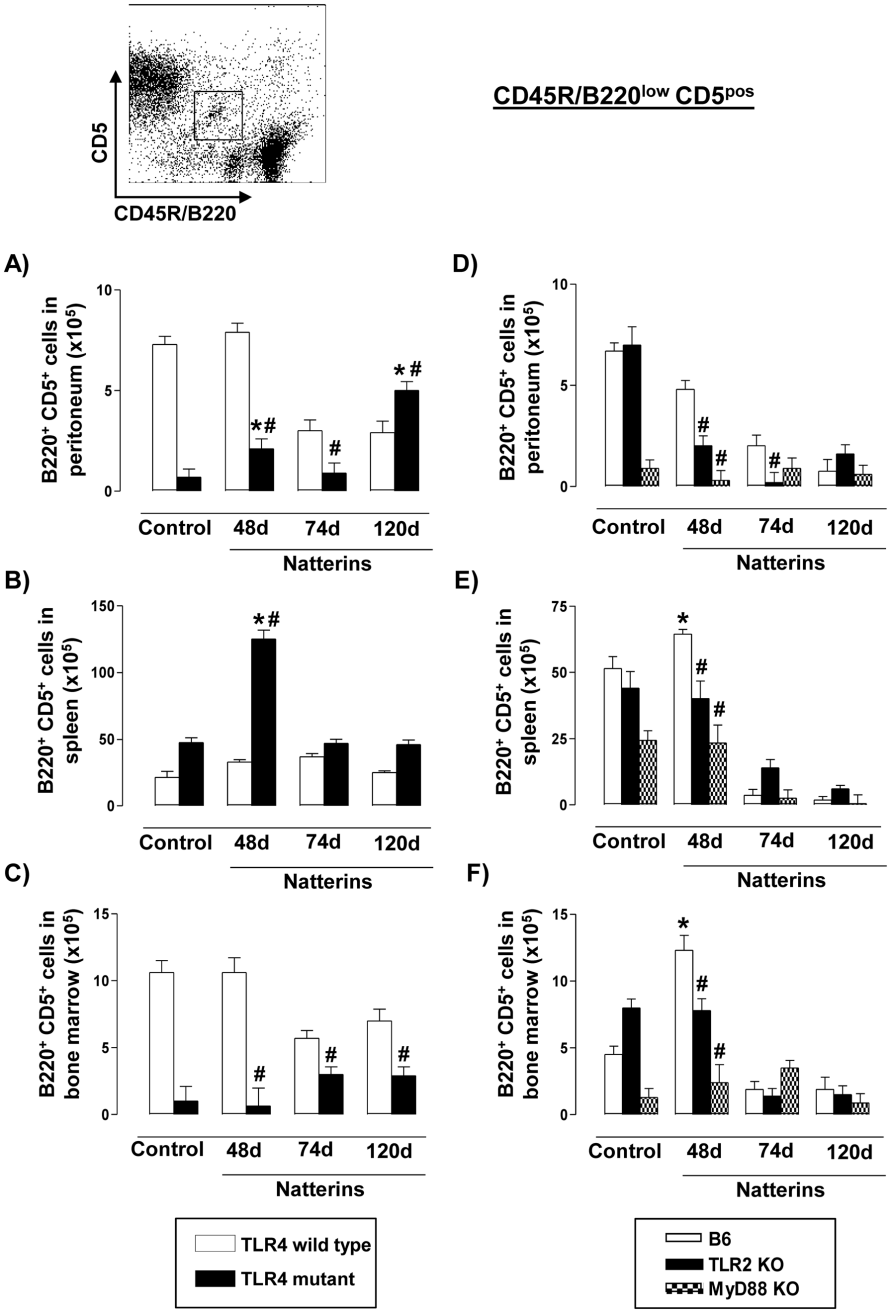
The transient expansion of B1a cells in spleen and bone marrow is positively mediated by TLR2 and MyD88 and negatively regulated by TLR4. A representative dot plot of B1a cells (B220^low^CD5^pos^) analyses is shown. Cells from peritoneum (A, D), spleen (B, E) and bone marrow (C, F) were obtained from TLR4 mutant (*left*) or from TLR2 *KO* and MyD88 *KO* (*right*) Natterins immunized mice after 48, 74 and 120 days. The bars representative of the absolute numbers of B220^low^CD5^pos^ cells were determined from total mononuclear cells by multiparametric flow cytometer using Rat IgG2ak PE-anti-mouse CD5, and Rat IgG2ak PerCP-Cy5-anti-mouse CD45R/B220. *p<0.05 compared to control mice; and ^#^ *p*<0.05 compared to *WT* mice immunized with Natterins.

First, we observed in the Figures **3** ***A***–**3** ***C*** that Natterins did not induce a chronic expansion of B1a cells into peritoneal cavity, spleen or in the BM of TLR4 *WT* mice (white bars), but in contrast this subpopulation was expanded in the peritoneal cavity of TLR4 mutant mice (black bars) mainly at 120 d (except at 74 d) and only at 48 d in spleen. TLR4 mutant mice did not expand B1a cells after immunization with Natterins in BM.

Second, in C57BL/6 *WT* mice (white bars) Natterins did not promote an expansion of the number of B1a cells (B220^low^CD5^pos^) in the peritoneal cavity (Fig. **3** ***D***), but only at early period of the chronic response in spleen (Fig. **3** ***E***) and in BM (Fig. **3** ***F***), compared with C57BL/6 non-immunized control mice. Our results showed that the absence of TLR2 or MyD88 determined the decrease in the absolute number of B1a cells in all compartments in the early phase compared with *WT* immunized-mice, indicative of positive regulation of early expansion of these cells by TLR2 and MyD88 signaling.

Together these results showed that the transient increased expansion of B1a cells at early periods of response to Natterins in spleen and BM seems to be positively mediated by mechanisms dependent on TLR2 and MyD88, moreover TLR4 is important to sustain the contraction of these cells in peritoneal cavity at the late stage of the immune response to Natterins.

### The persistence of B1b cells in bone marrow is negatively regulated by TLR2 and MyD88 signaling

**Figure 4:**
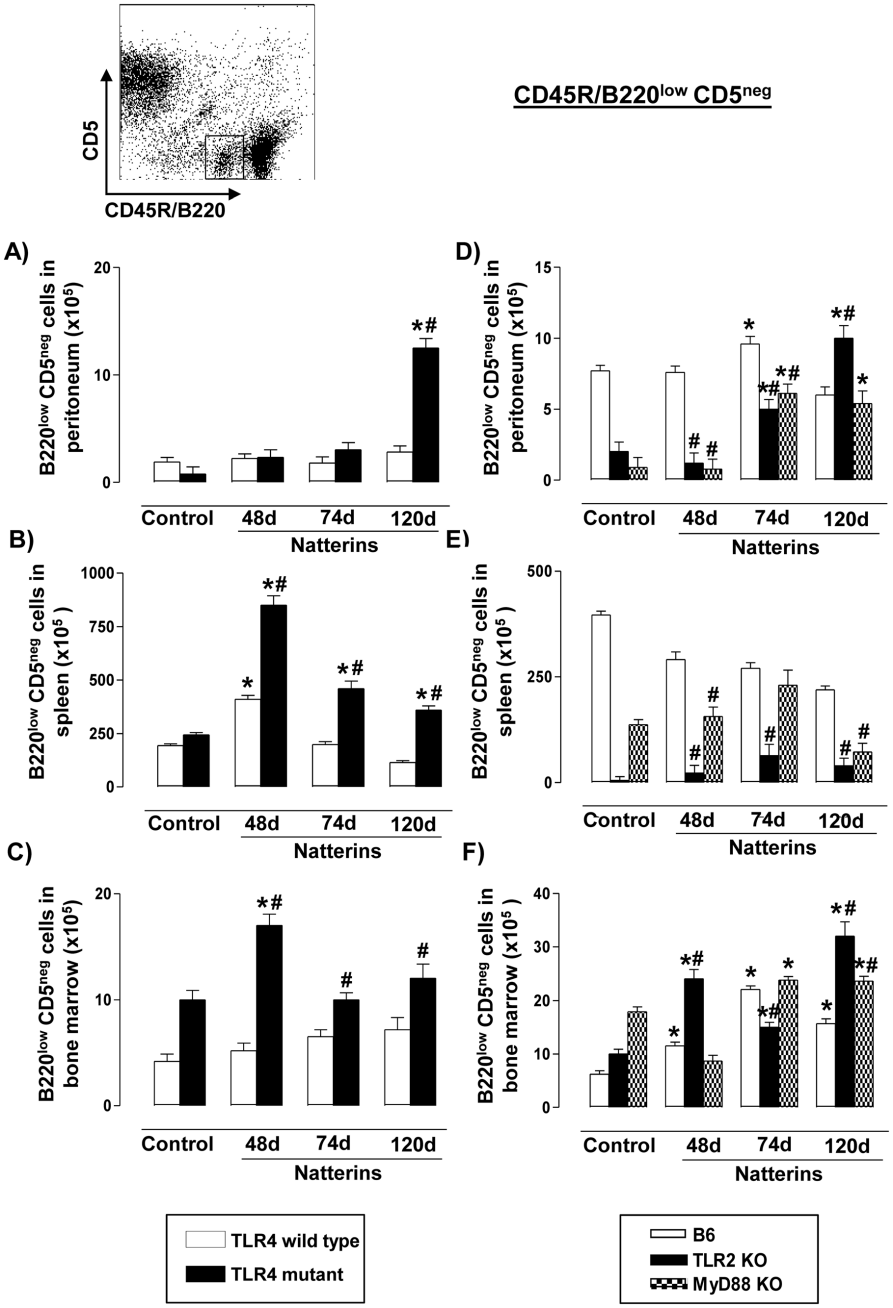
The persistence of B1b cells in bone marrow is negatively regulated by TLR2/TLR4 and MyD88 signals. A representative dot plot of B1b cells (B220^low^CD5^neg^) analyses is shown. Cells from peritoneum (A, D), spleen (B, E) and bone marrow (C, F) were obtained from TLR4 mutant (*left*) or from TLR2 *KO* and MyD88 *KO* (*right*) Natterins immunized mice after 48, 74 and 120 days. The bars representative of the absolute numbers of B220^low^CD5^neg^ cells were determined from total mononuclear cells by multiparametric flow cytometer using Rat IgG2ak PE-anti-mouse CD5, and Rat IgG2ak PerCP-Cy5-anti-mouse CD45R/B220. *p<0.05 compared to control mice; and ^#^ *p*<0.05 compared to *WT* mice immunized with Natterins.

Regarding B1b cells (B220^low^CD5^neg^) our results showed that Natterins did not induce the expansion of B1b cells into peritoneal cavity (Fig. **4** ***A***) or in the BM (Fig. **4** ***C***) of TLR4 *WT* mice (white bars), but in contrast this subpopulation was expanded in the spleen only at 48 d in TLR4 *WT* mice (Fig. **4** ***B***). In TLR4 mutant mice (black bars) Natterins induced an increased number of B1b cells only at 120 d in peritoneal cavity (Fig. **4** ***A***), and in entire course of the response in spleen (Fig. **4** ***B***), and only in the early phase in BM (Fig. **4** ***C***), indicating that TLR4 acts as negative regulator, mainly in the transient expansion of B1b cells in spleen.

In contrast, Natterins induced an increased expansion of B1b cells into peritoneal cavity (Fig. **4** ***D***) only at 74 d after immunization of the C57BL/6 *WT* mice that presented elevated number of these cells also in BM until 120 d (Fig. **4** ***F***). However, Natterins did not expand B1b cells in spleen of the C57BL/6 immunized-mice (Fig. **4** ***E***). After immunization with Natterins, TLR2 or MyD88 *KO* mice presented B1b cells until 120 d in peritoneal cavity and in BM, but not in spleen.

Together these results demonstrated that TLR4 is an important molecule for the retraction of the transient expansion of B1b cells observed in spleen, and that the longevity at late stage in BM is negatively regulated by TLR2-TLR4 and MyD88.

### TLR4 is important for the transient expansion of B2 cells in bone marrow, but TLR2 and MyD88 are crucial for their survival in this niche

**Figure 5:**
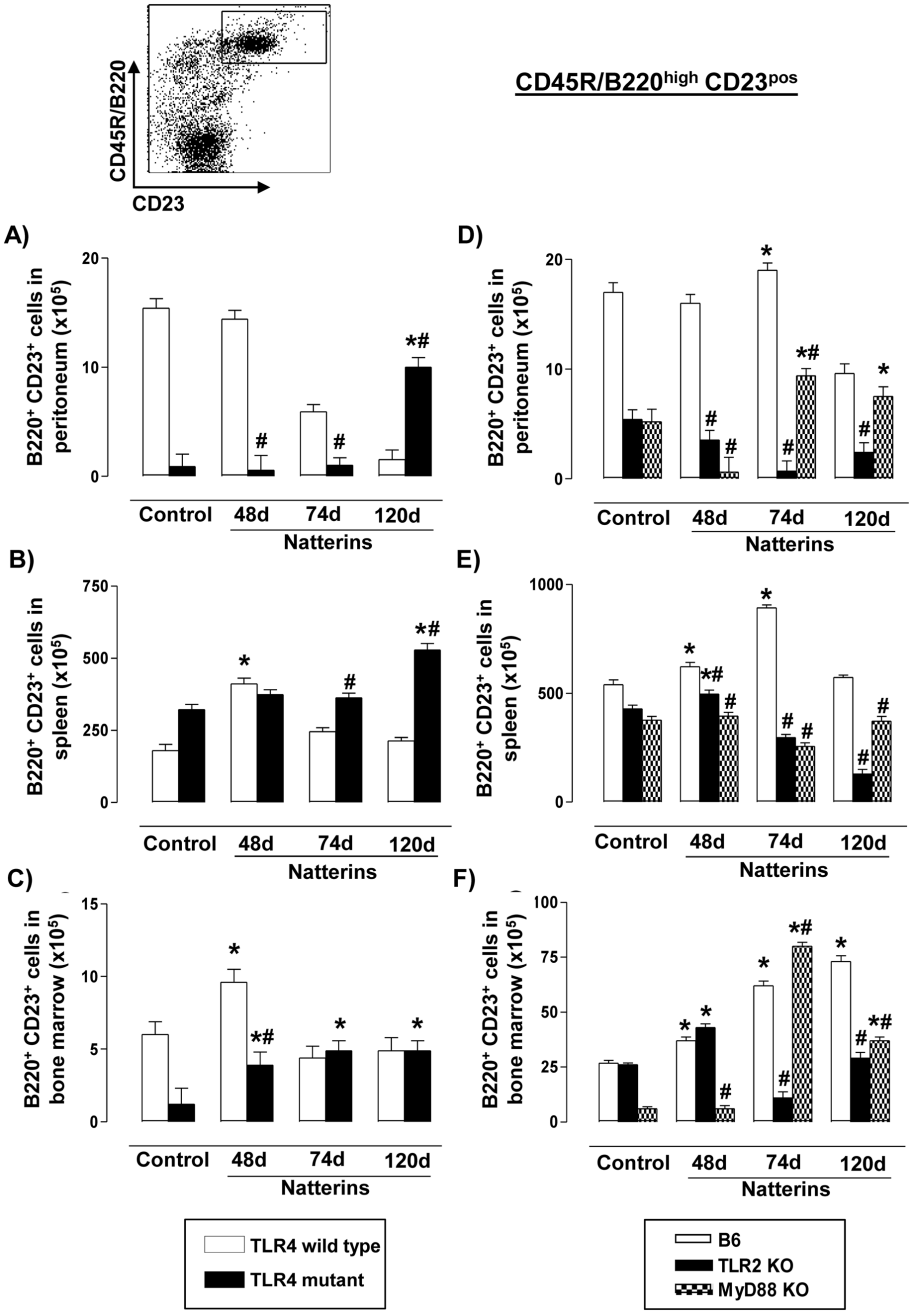
TLR4 is important for the transient expansion of B2 cells in bone marrow, but TLR2 and MyD88 are crucial for their survival in this niche. A representative dot plot of B2 cells (B220^high^CD23^pos^) analyses is shown. Cells from peritoneum (A, D), spleen (B, E) and bone marrow (C, F) were obtained from TLR4 mutant (*left*) or from TLR2 *KO* and MyD88 *KO* (*right*) Natterins immunized mice after 48, 74 and 120 days. The bars representative of the absolute numbers of B220^high^CD23^pos^ cells were determined from total mononuclear cells by multiparametric flow cytometer using Rat IgG2ak PE-anti-mouse CD23, Rat IgG2ak PerCP-Cy5-anti-mouse CD45R/B220. *p<0.05 compared to control mice; and ^#^ *p*<0.05 compared to *WT* mice immunized with Natterins.

Conventional B or B2 cells (B220^high^CD23^pos^) are the dominant population in spleen, re-circulate and are continually generated in the BM. After the recognition of antigens in proper help from T cells, B2 cells migrate to germinal centers where they proliferate and mature the affinity of specific Abs, differentiating into Bmem and ASC [4]. Our results showed that Natterins did not induce the expansion of B2 cells into peritoneal cavity (Fig. **5** ***A***), but only at early stage of the response in spleen (Fig. **5** ***B***) and BM (Fig. **5** ***C***) of C3H TLR4 *WT* immunized-mice (white bars) compared to non-immunized control mice. In TLR4 mutant mice (black bars) Natterins induced an increased number of B2 cells only at 120 d in peritoneal cavity and in spleen, and during the time course of the response in the BM, indicating that TLR4 exerts positive control in the early response in BM, and controls the retraction of B2 cells in late phase of response in peritoneal cavity.

In contrast to C3H, C57BL/6 *WT* Natterins immunized-mice showed a transient expansion of B2 cells into peritoneal cavity (Fig. **5** ***D***) and in spleen (Fig. **5** ***E***) until 74 d and these cells were maintained in BM until 120 d (Fig. **5** ***F***). In TLR2 *KO*, Natterins did not induce the expansion of B2 cells in peritoneal cavity (Fig. **5** ***D***) and only at early stage in spleen (Fig. **5** ***E***) and BM (Fig. **5** ***F***). MyD88 *KO* immunized-mice presented an increased number of B2 cells at late phase of the response in peritoneal cavity and BM, and did not present an expansion of B2 cells in spleen. Comparing the results obtained with TLR2 and MyD88 *KO* to C57BL/6 *WT* immunized-mice we observed that TLR2 and MyD88 positively regulate the transient expansion of B2 cells in peritoneal cavity and spleen, and the longevity in BM.

Together, these results show that TLR4 controls the retraction of B2 cells in late phase of response in peritoneal cavity, and the longevity in BM marrow is positively dependent on TLR2/MyD88.

### TLR2 dependent on MyD88 signals positively regulated memory B cells in spleen

The immune response to protein-containing antigens elicits quiescent Bmem. Multiple mechanisms have been shown or suggested to contribute to the persistence Bmem, generating several opposing concepts, e.g. their antigen dependent versus antigen-independent maintenance and the contribution of resting versus continuously cycling cells to the memory B-cell pool [38]. To confirm the role of TLR4, TLR2 and MyD88 signaling on the regulation of the persistence Bmem, we evaluated all compartments for the presence of B220^pos^Ig^pos^CD19^pos^ in TLR4 mutant, TLR2 *KO* and MyD88 *KO* mice immunized with Natterins.

**Figure 6:**
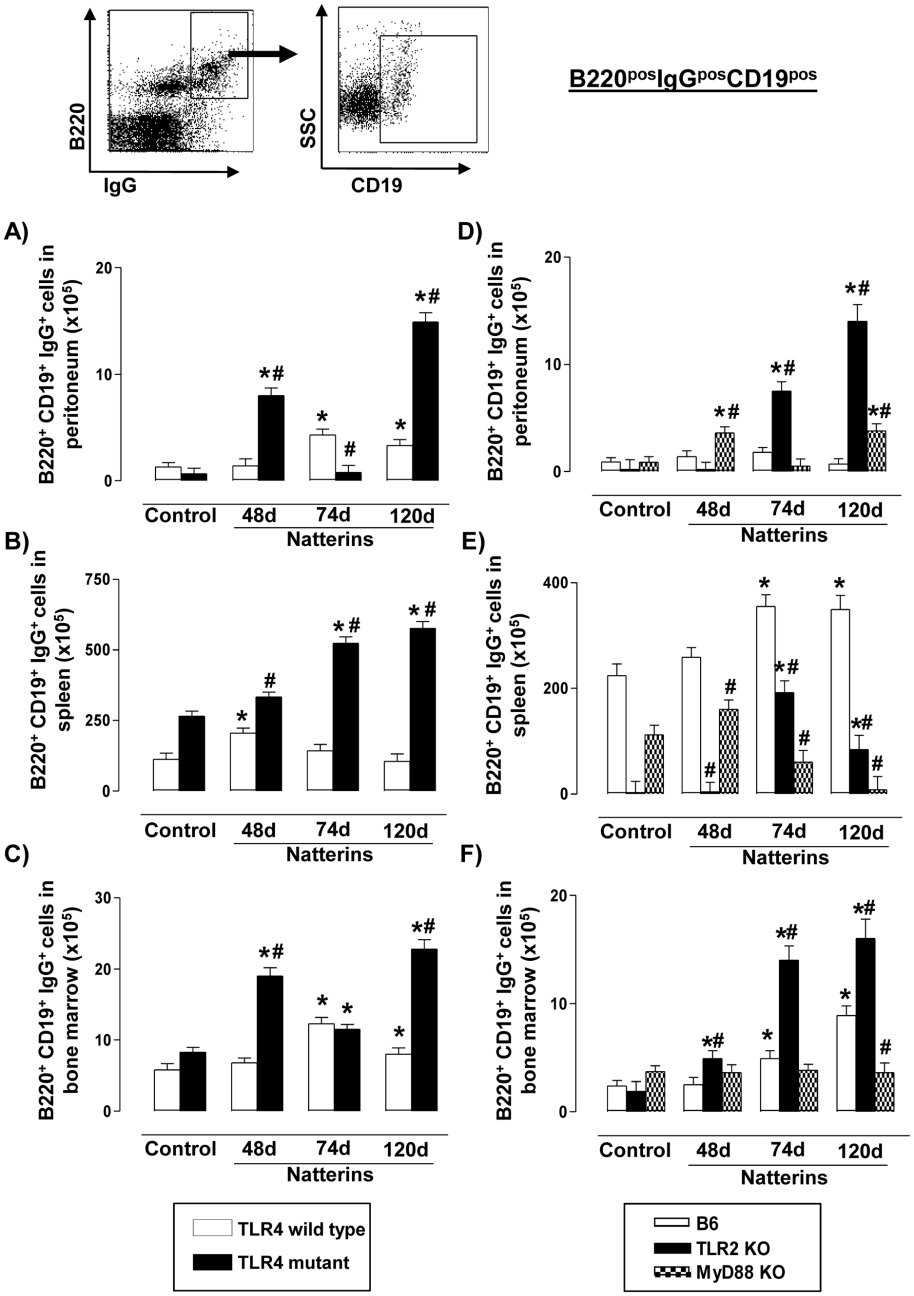
TLR2 dependent on MyD88 positively regulates Bmem in spleen and TLR4 negatively regulates in all compartments. A representative dot plot of Bmem cells (CD19^pos^ in B220^pos^Ig^pos^ gated cells) analyses is shown. Cells from peritoneum (A, D), spleen (B, E) and bone marrow (C, F) were obtained of TLR4 mutant (*left*), TLR2 *KO* and MyD88 *KO* (*right*) Natterins immunized mice after 48, 74 and 120 days. The bars representative of the absolute numbers of B220^pos^CD19^pos^Ig^pos^ cells were determined from total mononuclear cells by multiparametric flow cytometer using Armenian hamster IgG1y2 FITC-anti-mouse CD19, Goat IgG2bk PE-anti-mouse Ig (specific for IgG1, IgG2a, IgG2b and IgG3), Rat IgG2ak PerCP-Cy5-anti-mouse CD45R/B220.*p<0.05 compared to control mice; and ^#^ *p*<0.05 compared to *WT* mice immunized with Natterins.

We observed in the peritoneal cavity (Fig. **6** ***A***) and in BM (Fig. **6** ***C***) of TLR4 *WT* mice a persistent expansion of Bmem in response to Natterins, but in spleen these cells were transiently observed (Fig. **6** ***B***). In addition, TLR4 mutant mice developed after immunization with Natterins a intensified and sustained response of Bmem in all compartments. These results together demonstrated that TLR4 down-regulates the chronic retention of Bmem in the immune response to Natterins.

C57BL/6 *WT* (white bars) mice immunized with Natterins presented similar number of cells compared to non-immunized control mice into peritoneum (Fig. **6** ***D***), but these mice showed persistent and elevated number of Bmem in BM (Fig. **6** ***F***) and mainly in spleen (Fig. **6** ***E***). TLR2 *KO* mice (black bars) in contrast, showed in all compartments an increased number of Bmem until 120 d, after immunization with Natterins. The absence of MyD88 resulted in an accumulation of Bmem in the peritoneal cavity, whereas in the spleen the absence of MyD88 resulted in a drastic reduction of Bmem (striped grey bars).

These findings showed that TLR2 dependent on MyD88 signals positively in the longevity of Bmem in spleen. Moreover, TLR2 and TLR4 play a negative regulation dependent on MyD88 signaling in the maintenance of Bmem in peritoneal cavity, and independent in the BM.

### The chronic retention of ASC B220^pos^ in the peritoneum is dependent on TLR4 signaling

The cellular differentiation of Bmem into ASC has not been completely elucidated, but a hierarchical model of differentiation has been proposed: activated B cells progressively acquire increasing levels of CD138 and decreasing levels of CD45R/B220 to finally arrive at ASC with B220^neg^ phenotype [39], [40]. Here, we examined the population of ASC with a positive expression of B220 (B220^pos^CD43^pos^CD138^pos^) and ASC with negative expression of B220 (B220^neg^CD43^pos^CD138^pos^) in *WT*, TLR4 mutant, TLR2 *KO* and MyD88 *KO* mice immunized with Natterins.

**Figure 7:**
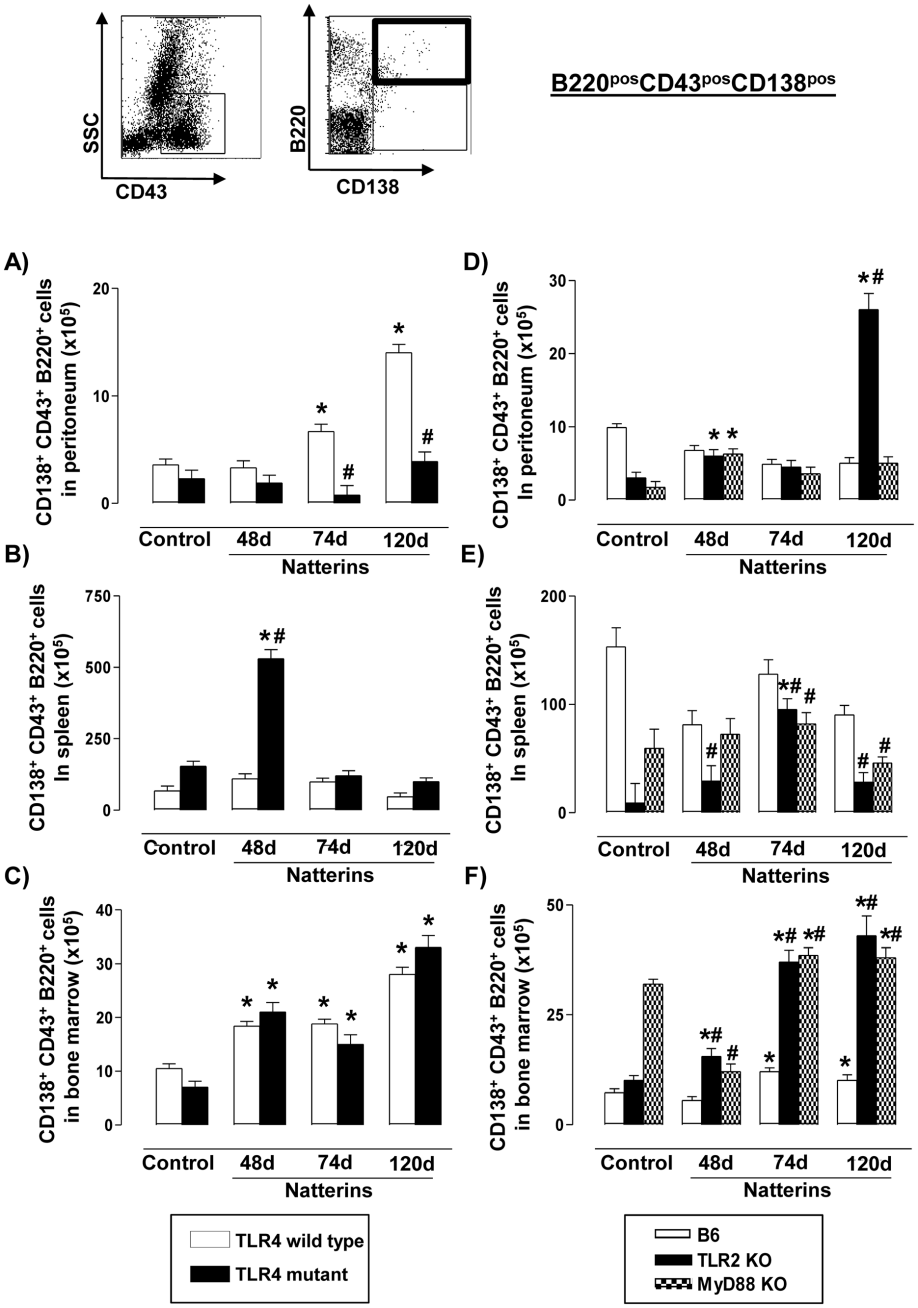
The chronic retention of ASC B220^pos^ in the peritoneum is dependent on TLR4 signals. A representative dot plot of ASC B220^pos^ (B220^pos^CD138^pos^ from CD43-positive gated cells) analyses is shown. Cells from peritoneum (A, D), spleen (B, E) and bone marrow (C, F) were obtained of TLR4 mutant (*left*), TLR2 *KO* and MyD88 *KO* (*right*) Natterins immunized mice after 48, 74 and 120 days. The bars representative of the absolute numbers of B220^pos^ CD43^pos^CD138^pos^ cells were determined from total mononuclear cells by multiparametric flow cytometer using Rat IgG2ak PerCP-Cy5-anti-mouse CD45R/B220, Rat IgG2ak FITC-anti-mouse CD43, and Rat IgG2ak PE-anti-mouse CD138. *p<0.05 compared to control mice; and ^#^ *p*<0.05 compared to *WT* mice immunized with Natterins.

Our results showed that Natterins sustain during chronic response the presence of ASC B220^pos^ in the peritoneal cavity (Fig. **7** ***A***) and in BM (Fig. **7** ***C***) in TLR4 *WT* mice, in contrast to the basal number in the spleen compared with control mice (Fig. **7** ***B***). Comparing to C3H *WT* immunized-mice, the absence of TLR4 resulted in a drastic decrease of these cells in the inflamed peritoneal cavity, suggesting a positive regulation by TLR4. In recent periods in the spleen, ASC B220^pos^ appear to be negatively regulated by TLR4. The absence of TLR4 did not affect the profile of response of ASC B220^pos^ in BM.

C57BL/6 *WT* (white bars) mice immunized with Natterins presented similar number of ASC B220^pos^ compared to non-immunized control mice into peritoneum (Fig. **7** ***D***) and in spleen (Fig. **7** ***E***), but not in BM where the number of cells was significant increased after 74 d (Fig. **7** ***F***). The absence of TLR2 in immunized-mice reflected in a drastic increase of ASC B220^pos^ at 120 d in the peritoneum, and during entire course of response in BM, with a transient appearance of these cells at 74 d in spleen. MyD88 *KO* mice developed a similar number of cells compared to C57BL/6 *WT* immunized-mice in peritoneal cavity and spleen, but only in BM an increased number of these cells were observed.

Together, our results showed that during the anti-Natterins immune response, the positive regulation of the maintenance of ASC B220^pos^ in the peritoneum occurs dependently on TLR4 signaling, while in BM TLR2 and MyD88 are important for negative regulation.

### TLR2, TLR4 and MyD88 signaling can sustain ASC B220^neg^ into peritoneal cavity

**Figure 8:**
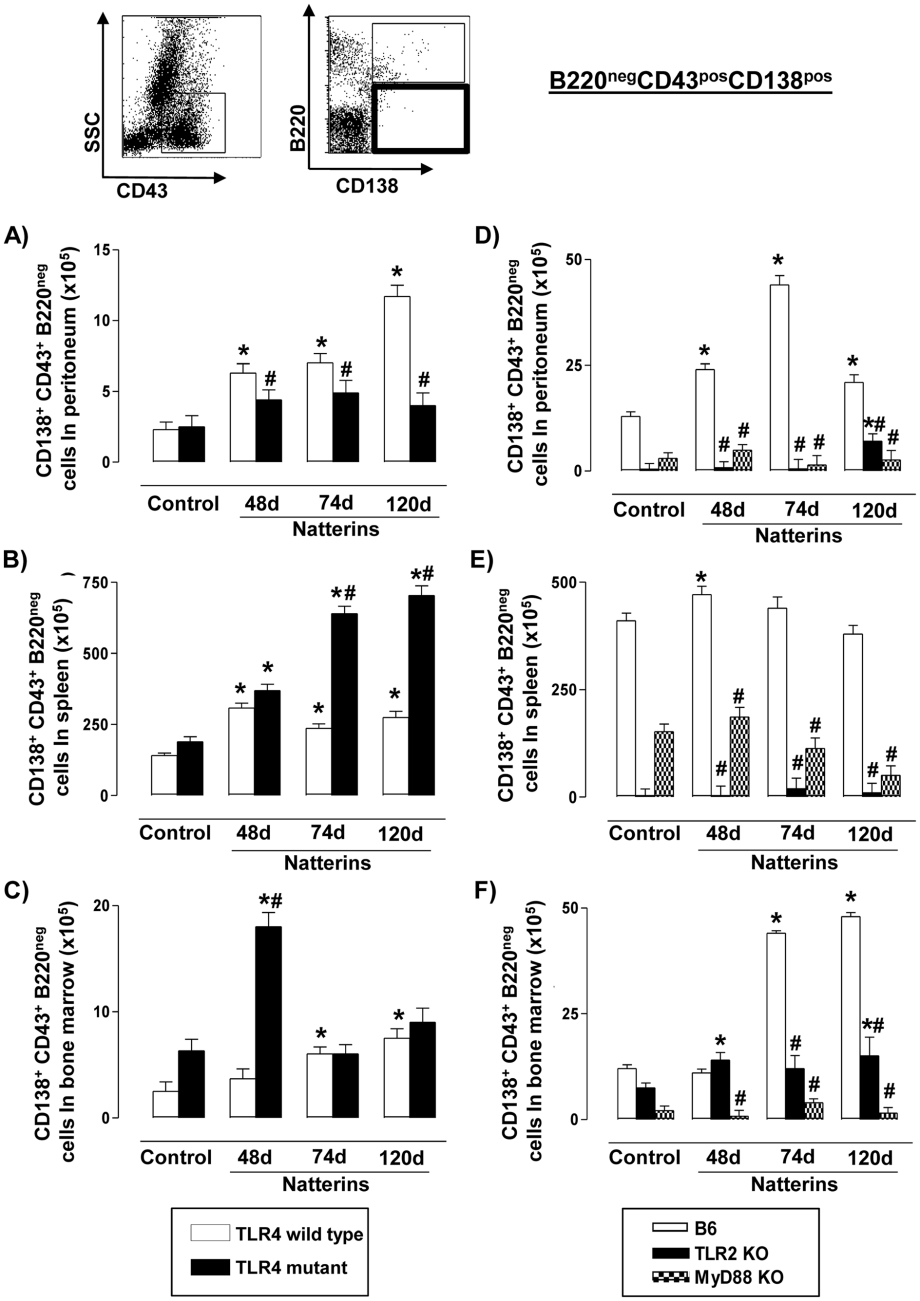
TLR2, TLR4 and MyD88 signaling can sustain ASC B220^neg^ in peritoneal cavity. A representative dot plot of ASC B220^neg^ (B220^neg^CD138^pos^ from CD43-positive gated cells) analyses is shown. Cells from peritoneum (A, D), spleen (B, E) and bone marrow (C, F) were obtained of TLR4 mutant (*left*), TLR2 *KO* and MyD88 *KO* (*right*) Natterins immunized mice after 48, 74 and 120 days. The bars representative of the absolute numbers of B220^neg^CD43^pos^CD138^pos^ cells were determined from total mononuclear cells by multiparametric flow cytometer using Rat IgG2ak PerCP-Cy5-anti-mouse CD45R/B220, Rat IgG2ak FITC-anti-mouse CD43, and Rat IgG2ak PE-anti-mouse CD138. *p<0.05 compared to control mice; and ^#^ *p*<0.05 compared to *WT* mice immunized with Natterins.

Our results showed that Natterins induce in C3H TLR4 *WT* immunized-mice (white bars) a sustained expansion of ASC B220^neg^ into peritoneal cavity (Fig. **8** ***A***), spleen (Fig. **8** ***B***) and BM (Fig. **8** ***C***) compared to non-immunized control mice. In TLR4 mutant mice (black bars) Natterins also did not induce expansion of ASC B220^neg^ in peritoneal cavity, but only in spleen, and transiently in the BM. Comparing the chronic response in both type of immunized-mice (*WT* and mutant), we observe that in peritoneal cavity the number of these cells in mutant mice was lower comparing to *WT* immunized-mice. But in spleen, the number of ASC B220^neg^ was higher, indicating that TLR4 exerts positive control in peritoneal cavity but negative control in the persistence in spleen.

C57BL/6 *WT* mice showed an increased number of ASC B220^neg^ into peritoneal cavity (Fig. **8** ***D***) and in BM (Fig. **8** ***F***) until 120 d, but a transient elevation in the number of these cells in spleen was observed (Fig. **8** ***E***). In TLR2 *KO*, Natterins only induced the expansion of ASC B220^neg^ in peritoneal cavity and BM at late stages of the response, but not in spleen. Natterins were not able to induce an expansion of the number of ASC B220^neg^ in MyD88 *KO* immunized-mice. Comparing the results obtained with TLR2 and MyD88 *KO* to C57BL/6 *WT* immunized-mice we observed that TLR2 and MyD88 positively regulate ASC B220^neg^ cells in all compartments.

Our results together suggest that the survival of ASC B220^neg^ into all compartments for long periods depends on TLR2 and MyD88. Also TLR4 co-participates in the maintenance of ASC B220^neg^ into the peritoneal cavity. In contrast, TLR4 acts negatively regulating these cells into spleen.

### The role of B lymphocytes recirculation through lymphoid organs and final mobilization in specific organs and tissues

Fingolimod (FTY720-P) is a pro-drug with immunosuppressive effects elicited following its phosphorylation by sphingosine kinase 2 that binds G protein-coupled sphingosine 1-phosphate (S1P) receptor 1 (SP1R) causing their internalization and degradation. Fingolimod blocks the sphingosine-1-phosphate gradient controlled lymphocyte egress from the lymph nodes and therefore reduces the peripheral lymphocyte count [41].

**Figure 9:**
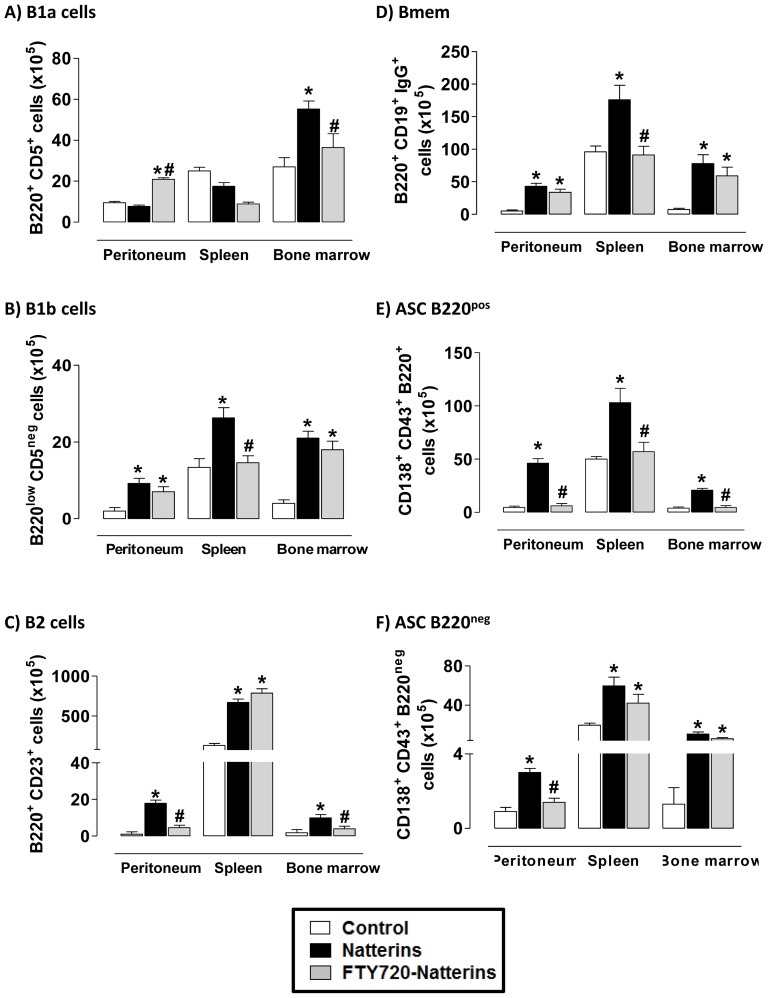
The impaired lymphocyte egress from the secondary lymph organs controls the migration of innate-like B cell and memory B cell for peripheral tissues. Cells from peritoneum, spleen and bone marrow of Natterins-immunized BALB/c mice treated or not with FTY720 were obtained at day 28. The bars representative of the absolute numbers of B1a cells (B220^+^CD5^+^) (***A***), B1b cells (B220^low^CD5^neg^) (***B***), B2 cells (B220+CD23+) (***C***), Bmem (B220^+^CD19^+^IgG^+^) (***D***) ASC B220^pos^ (CD138^+^CD43^+^B220^+^) (***E***), ASC B220^neg^ (CD138^+^CD43^+^B220^neg^) (***F***) were determined from total mononuclear cells by multiparametric flow cytometer using Rat IgG2ak PE-anti-mouse CD5, Rat IgG2ak PerCP-Cy5-anti-mouse CD45R/B220, and Rat IgG2ak PE-anti-mouse CD23, Armenian hamster IgG1y2 FITC-anti-mouse CD19, Goat IgG2bk PE-anti-mouse IgG (specific for IgG1, IgG2a, IgG2b and IgG3), Rat IgG2ak FITC-anti-mouse CD43, and Rat IgG2ak PE-anti-mouse CD138. *p<0.05 compared to control mice; and ^#^ *p*<0.05 compared to Natterins-immunized mice without treatment.

To test the possible role of SP1R in B lymphocytes recirculation through lymphoid organs and final mobilization in specific organs and tissues, we treated Natterins-immunized mice with FTY720 before immunization procedures. In Figure **9**, we observed at 28 d that the secondary immune response induced by Natterins in BALB/c mice is characterized by the presence of B1a cells only in BM (Fig. **9** ***A***) and B1b and B2 cells in all compartments (Fig. **9** ***B*** and **9** ***C***). Also B2 is the predominant subtype of cells at this time of response against Natterins, and the splenic environment sustained the higher number of innate-like B cells. When Natterins-immunized mice were treated at days 0, 7 and 21 with FTY720, a functional antagonist of S1PR, the innate B cell compartment was modified by the reduced egress from secondary lymph organs. The treatment induced an augmented number of B1a cells in peritoneal cavity, but reduced these cells at basal number into BM (Fig. **9** ***A***). Also, we observed that FTY720 treated mice presented reduced number of B1b cells in spleen (Fig. **9** ***B***); and reduced number of B2 cells in peritoneal cavity and BM (Fig. **9** ***C***).

The memory B cell compartment was also modified by treatment with the functional antagonist of S1PR, FTY720. In Figure **9** ***D*** the data show that the mobilization of Bmem into the peritoneal cavity or into BM was not changed after FTY720 treatment, but in contrast the retention into splenic environment was reduced at basal levels. In addition, the mobilization of ASC B220^pos^ into peritoneal cavity or BM and the retention of these cells into spleen were dependent on S1PR signals derived from lymph or blood circulation. Finally, the mobilization of ASC B220^neg^ into peritoneal cavity was also dependent on lymph- or blood-derived S1P signals. The mobilization of ASC B220^neg^ into BM and the retention into spleen were not modified by FTY720 treatment.

The chemokine receptor CXCR4 is abundant on naive lymphocytes and it can promote cell entry to LNs. During the maturation process of memory B cells the levels of CXCR4 dramatically increase (beginning in naive , activated, light zone GC, and reaching plasmablast and plasma cell stages). To explore whether the accumulation of ASC (B220^pos^ and B220^neg^) in inflamed peritoneal tissue may be a consequence of egress from secondary lymph organs we evaluated the expression levels of CXCR3 and CXCR4 in memory B cells of Natterins-immunized mice treated with FTY720.

**Figure 10:**
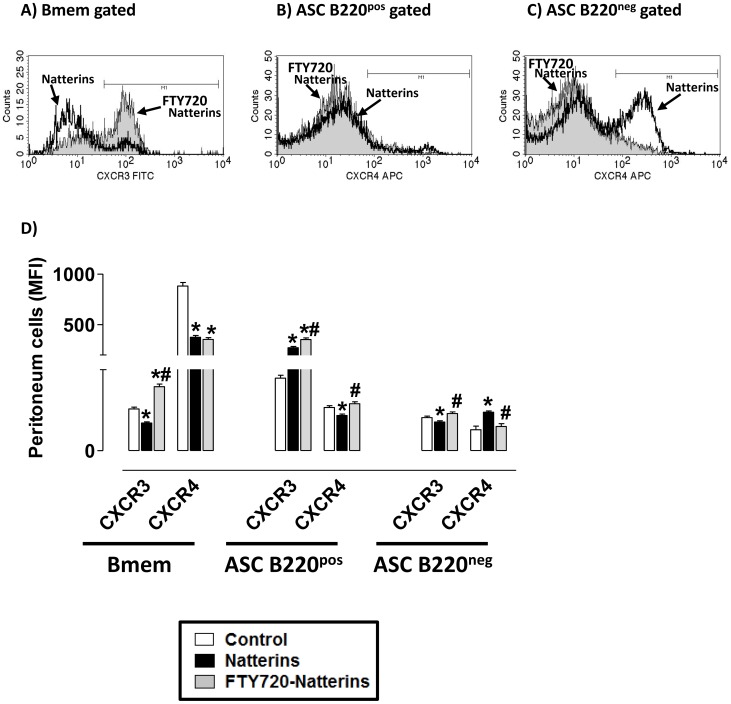
The egress of lymphocytes from secondary lymph organs modulates the balance of CXCR3/CXCR4 expression in B cell memory compartment. Expression of CXCR3 and CXCR4 on Bmem, ASC B220^pos^ and ASC B220^neg^ in peritoneal inflamed tissue at 28 d after FTY720 treatment of Natterins immunized-mice. Representative histograms gated on memory B cell compartments show mean fluorescence intensity (MFI). *p<0.05 compared to control mice; and ^#^ *p*<0.05 compared to Natterins-immunized mice without treatment.

In the Figure **10**, compared with memory B cells from control-mice, we can observe diminished levels of CXCR3 and CXCR4 in Bmem from Natterins-immunized mice, elevated levels of CXCR3 and diminished levels of CXCR4 in ASC B220^pos^, and in contrast diminished levels of CXCR3 and elevated levels of CXCR4 in ASC B220^neg^ cells. Moreover, our data show that after FTY720 treatment, the expression of CXCR3 was augmented in all memory B cells, indicative of a negative regulation of lymph- or blood-S1PR signal. In addition, the treatment reduced to basal levels the CXCR4 expression in ASC B220^neg^ cells, and in contrast induced augmented expression of CXCR4 in ASC B220^pos^ cells.

The presence of cytokines-producing cells was evaluated by flow cytometry on splenic CD4^pos^ T cells, Ly6C^pos^ macrophages and CD11b^pos^CD11c^pos^ DC from Natterins-immunized mice treated or not with FTY720 obtained at day 28, after their restimulation with PMA and ionomycin in *in vitro* system (Fig. **S1**). Splenic cells from Natterins-immunized mice without treatment presented remarkable proportions of IL-17-producing CD4^pos^ T cells (1.2 fold) and IL-13-producing DC (2.0 fold), compared with control-mice. Also Natterins induced an increased proportion of IFN-γ-producing DC (2.2 fold), whereas cytokines-producing Ly6C^pos^ macrophages were virtually undetectable. After treatment with FTY720, restimulated splenic cells presented a stronger cytokine-secreting capacity compared with cells from non-treated mice. FTY720 splenic cells presented an augment in the proportion of IFN-γ- and IL-17A-producing CD4^pos^ T cells, in the IFN-γ-, IL-17A-, and IL-13-producing Ly6C^pos^ macrophages, and in the IFN-γ- and IL-13-producing DC.

## Discussion

Ligation of TLRs with their cognate ligands leads to a cytosolic signaling cascade beginning with the recruitment of the proximal adapters MyD88 or TRIF and subsequent initiation of pro-inflammatory gene transcription through pathways including NF-κB and mitogen-activated protein kinase (MAPK) cascades [42]. Recognition of pathogen-associated molecules by TLRs expressed on “classic” innate cells, such as DC and macrophages, triggers their maturation leading to initiation of antigen-specific adaptive immune responses through T cell activation. Furthermore, direct signals through TLRs expressed on B cells play an important role in the activation and optimal Abs production to T-dependent antigens [18].

Several B cell subsets express TLRs [31] and can be activated via TLR ligands that result in robust proliferation and Abs secretion, rapid up-regulation of Blimp-1 and XBP-1_S_, induction of CD138, and morphological changes compatible with acquisition of a high secretory capacity [32]. TLRs mediated signals synergize with self-antigen-mediated BCR signals to stimulate activation of self-reactive B cells [43], and B cell activation was severely reduced when the mice were deficient in TLRs signaling [44].

**Figure 11:**
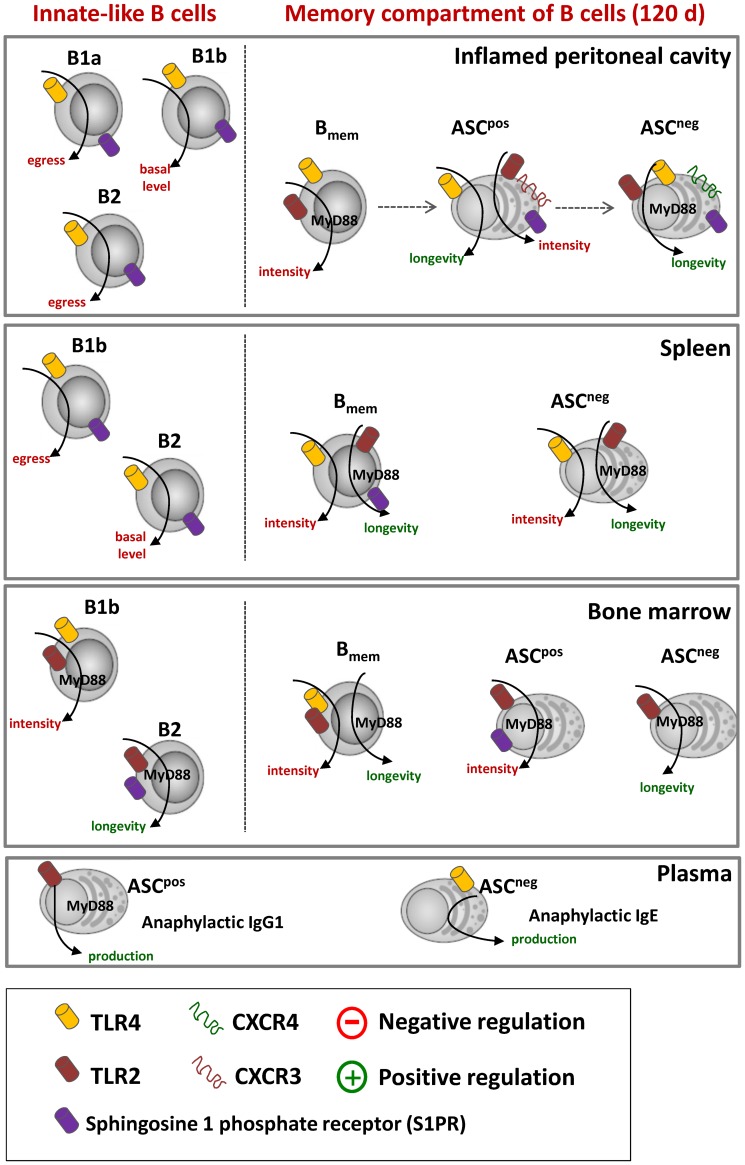
The importance of the integration of signaling pathways downstream of SP1R and TLRs in modulating the maintenance of ASC in peritoneal cavity. We propose that during secondary immune response against Natterins, after some period in the lymphoid organs, S1PRs are required for TLR-induced peritoneal B1a and B2 cell egress and also for medullar B2 longevity; and that the longevity of Bmem in splenic niche, the intensity of ASC B220^pos^ into peritoneal cavity and BM, and mainly the longevity of ASC B220^neg^ into inflamed peritoneal tissue is strong supported by CXCR4 expression dependent on SP1R expression in B lymphocytes and its recirculation through lymphoid organs.

All abovementioned data confirm the sufficient role of TLRs stimulation for eliciting a B cell activation and effector functions, but the contribution of TLRs into memory B cell compartment differentiation and survival is still not clear. Here we demonstrated in an *in vivo* model that chronic humoral response induced by Natterins is accompanied by the long-term innate-like B cells as B1b and B2 in the BM, and Bmem and ASC B220^neg^ in all compartments; and ASC B220^pos^ only in the peritoneum and BM. Further we highlighted the involvement of TLRs in the control of the overall magnitude of response induced by the proteases Natterins, in the relationship of B cells migration and differentiation and the persistence of distinct subtype of B cells into specific tissue niches. Together, our results clearly indicate that during secondary immune response against Natterins, after some period in the lymphoid organs, S1PRs are required for TLR-induced peritoneal B1a and B2 cell egress and also for medullar B2 longevity; and that the longevity of Bmem in splenic niche, the magnitude of ASC B220^pos^ into peritoneal cavity and BM. The present study confirms that S1PRs are required for triggering ASC B220^pos^ into peritoneal cavity and BM and for directing the migration of ASC B220^neg^ into inflamed peritoneal cavity (Fig. **11**).

Initially, our findings demonstrated that the control of the production of anaphylactic Abs induced by Natterins as IgG1 is dependent on TLR2 and MyD88 signaling, and that TLR4 acts as adjuvant accelerating the synthesis of high affinity-IgE. However, we also showed that TLR4 mutant as well as TLR2 *KO* mice sustain the expansion of Bmem, ASC B220^pos^ and ASC B220^neg^ in chronic periods (at 120 d) of the Natterins-response, suggesting that the modulation of anaphylactic Abs response is not related with the absence of TLR2/4 expression in B cells. As DC also express TLRs [45], the effect of TLR deficiency on Abs production could be explained by an indirect effect on DCs.

Second, we have demonstrated that the TLR4 derived signals (MyD88-independent) modulate the migration of innate-like B cells as B1a and B2 out of the peritoneal cavity, and the emigration from the spleen of B1b and B2 cells. Activation-induced redistribution of body cavity B1 cells seems to be a common response in various disease models, it is probable that multiple innate immune signals can induce changes in B1 cell mobility, possibly by regulating their expression of integrins and chemokine receptors. Our data presented here corroborate the findings by others that showed that TLRs induce a massive egress of innate-like B1 cells from the peritoneal cavity to other lymphoid organs [46], [47]. In addition, the migration out of the peritoneum of B1 cells with subsequent differentiation into IgM-secreting ASC in spleen could be expected, once B1a cells express higher levels of Blimp-1 [48] and the differentiation process is done outside of peritoneal cavity [49].

Furthermore, mice that carrying a mutation in TLR4 showed an accumulation of Bmem in peritoneal cavity at chronic periods of Natterins response, accompanied by a drastic decrease in both ASC B220^pos^ and ASC B220^neg^ in this inflamed tissue. These data reinforce the idea of the hierarchical relationship between the two types of memory B cells (continuous differentiation of ASC B220^neg^ from and ASC B220^pos^ and Bmem) when show that TLR4 signals are sufficient to activate Bmem to differentiate into ASC *in vivo*. Literature data demonstrated that agonists of TLRs are sufficient to induce the expression of Blimp-1 by B cells differentiating them into ASC in an *in vitro* system [7], [19], [22], [23].

In addition, we demonstrated that TLR4 derived signals (MyD88-independent) modulate the emigration from the spleen of Bmem as well as ASC B220^pos^. TLR2 triggered to the egress from the peritoneum of Bmem (MyD88-dependent) and ASC B220^pos^ (MyD88-independent). The retention and migratory signals that orchestrate the movements of germinal center B cells remain only partially understood, although our data sustain that earlier events on memory B cells differentiation, after secondary lymph organs influx and egress, may be the key to determining peripheral localization of innate-like B cells and memory B cells as ASC B220^pos^ and ASC B220^neg^.

The present studies confirm that the longevity of ASC B220^neg^ into inflamed peritoneal cavity induced by Natterins is strong supported by up-regulated CXCR4 expression dependent on SP1R signals in B lymphocytes and its recirculation through lymphoid organs (Fig. **11**). CXCL2 a ligant of CXCR4 may influence migration of activated B cells within the GC and ASC survival [50], [51], and expression of receptors for CXCR4 and CXCR5 on the GC B cells may direct emigration or recycling and affinity maturation in the GC [52]–[54].

We reasoned that the release of both chemokines and cytokines during the primary induction of inflammation and secondary activation induced by the proteases Natterins, dependent on TLR activation, could promote the modulation of chemokine receptors expression that lead to B cell migration out of the peritoneal cavity and throughout of different zones of spleen. Next, we confirm that the systemic Th2/Th17 microenvironmental conditions induced by Natterins which sustain anaphylactic Abs production and CXCR4-positive ASC mobilization into specific compartments were changed by FTY720 treatment, leading to exacerbation of IL-17A/IFN-γ production with activation of M1 macrophages subtype.

Importantly, our results revealed both shared and divergent roles for TLR2 and TLR4 in the traffic and retention of the same subtype of cells (Bmem and ASC B220^pos^) in spleen or peritoneal cavity. Individual TLR activators can mediate selective responses, the same TLR can induce the exit of the one compartment and the subsequent attachment to another, and also the migration or attachment in the same compartment is dependent on different TLRs. TLR4 in particular can mediate the activation of IFN-β transcription, with subsequent autocrine activation of specific gene subsets [55], [56]. It has also been observed that only a subgroup of TLRs, including TLR2, but not TLR4, contain typical sequences allowing signaling via p85/p110 adapter proteins [57], although both TLR2 and TLR4 can activate signaling via Akt [58].

Consistent with a key role for TLRs in modulating B cell responsiveness, here we showed that TLR4 regulates the degree of expansion of Bmem in the peritoneum (MyD88-dependent) and in BM (MyD88-independent) as well as of ASC B220^neg^ in the spleen (MyD88-independent). TLR2 regulated the intensity of the expansion of Bmem (independent on MyD88) and ASC B220^pos^ (MyD88-dependent) in BM. Although TLRs signals can induce protective response against pathogens, and can potentially break tolerance and trigger autoimmune diseases, these same signals help to suppress immune responses and maintain self-tolerance. Some authors described various mechanisms for the TLR and IL-1R signaling negative regulation, including altered transcription, disruption of chromatin remodeling, altered cell surface receptor expression, expression of anti-inflammatory cytokines, and induction of negative regulators of TLR signaling [59]–[63].

Lastly, a striking characteristic of the response induced by Natterins is memory B cell compartment longevity. We showed that TLR4 signals sustain the longevity of ASC B220^pos^ (independent on MyD88) and ASC B220^neg^ into the peritoneum (MyD88-dependent) and that TLR2 MyD88-dependent signals support the persistence of B2 cells in BM, Bmem in the spleen and ASC B220^neg^ in peritoneum and BM. Collectively we conclude that activation of TLRs on various cell types (including direct action on B cells) during the course of anti-Natterins Th2 response signals via the MyD88 adapter molecule for the maintenance of Bmem in the spleen and ASC B220^neg^ in peritoneum and BM. And independent on signals derived from MyD88 and possibly via TRIF, TLRs sustain the longevity of ASC B220^pos^ in peritoneal cavity.

Furthermore, another aspect described here is a hierarchy in the longevity of memory B cell subtypes for the requirement of TLRs. Terminally differentiated ASC B220^neg^ required the cooperation of both signals through TLR2 and TLR4 via MyD88 for longevity in peritoneum, whereas Bmem required only TLR2/MyD88 to stay in spleen, and ASC B220^pos^ rested in peritoneum dependent on TLR4.

ASC are terminally differentiated and continue secreting Abs without antigenic stimulation in the BM that provides the necessary environment for their longevity. The transcription factors that are generally required for ASC differentiation are Blimp-1, interferon regulatory factor 4 (IRF4) and X-box binding protein 1 (Xbp1) [21], [22], [64], [65]. Remarkably, studies of mice deficient in a TLR4 downstream signaling molecule, MyD88, demonstrated that MyD88-deficient B cells also exhibited enhanced Bcl-6 expression and diminished Blimp-1 expression [18] and defects in their longevity.

In conclusion, the understanding of the requirement of TLR/MyD88 and the co-participation of lymph- or blood-derived S1PR signals for the *in vivo* differentiation and survival for long time of memory B cells and especially of the long-lasting subtype (ASC) induced by proteases derived from venomous fish with kininogenase activity allow us a further clarification of the role of proteases in the development and maintenance of chronic Th2 disorders.

## Supporting Information

Figure S1 **Natterins induce the production of IL-13 and IL-17A by splenic cells.** Spleen cells were restimulated for 16 h at 37°C, 5% CO_2_ with a cell stimulation cocktail containing PMA and ionomycin in the presence of brefeldin A and monesin. Subsequently, after washing and fixation, different subtypes of cells as CD4^pos^ T cells, Ly6C^pos^ macrophages and CD11b^pos^CD11c^pos^ DC were assessed and for intracellular content of IFN-γ, IL-17A, IL-4, IL-13 and IL-10. The percentages of viable positive-cells that produce cytokine in response to restimulation in culture are shown in tables. *p<0.05 compared to control mice; and ^#^ *p*<0.05 compared to Natterins-immunized mice without FTY720 treatment.
(TIFF)
