# Supplementary figures and images for: TLR2, TLR4 and the MyD88 Signaling Are Crucial For the In Vivo Generation and the Longevity of Long-Lived Antibody-Secreting Cells
Source: PLoS One. 2013 Aug 5;8(8):e71185. doi: 10.1371/journal.pone.0071185 (PMC3733974; doi:10.1371/journal.pone.0071185)

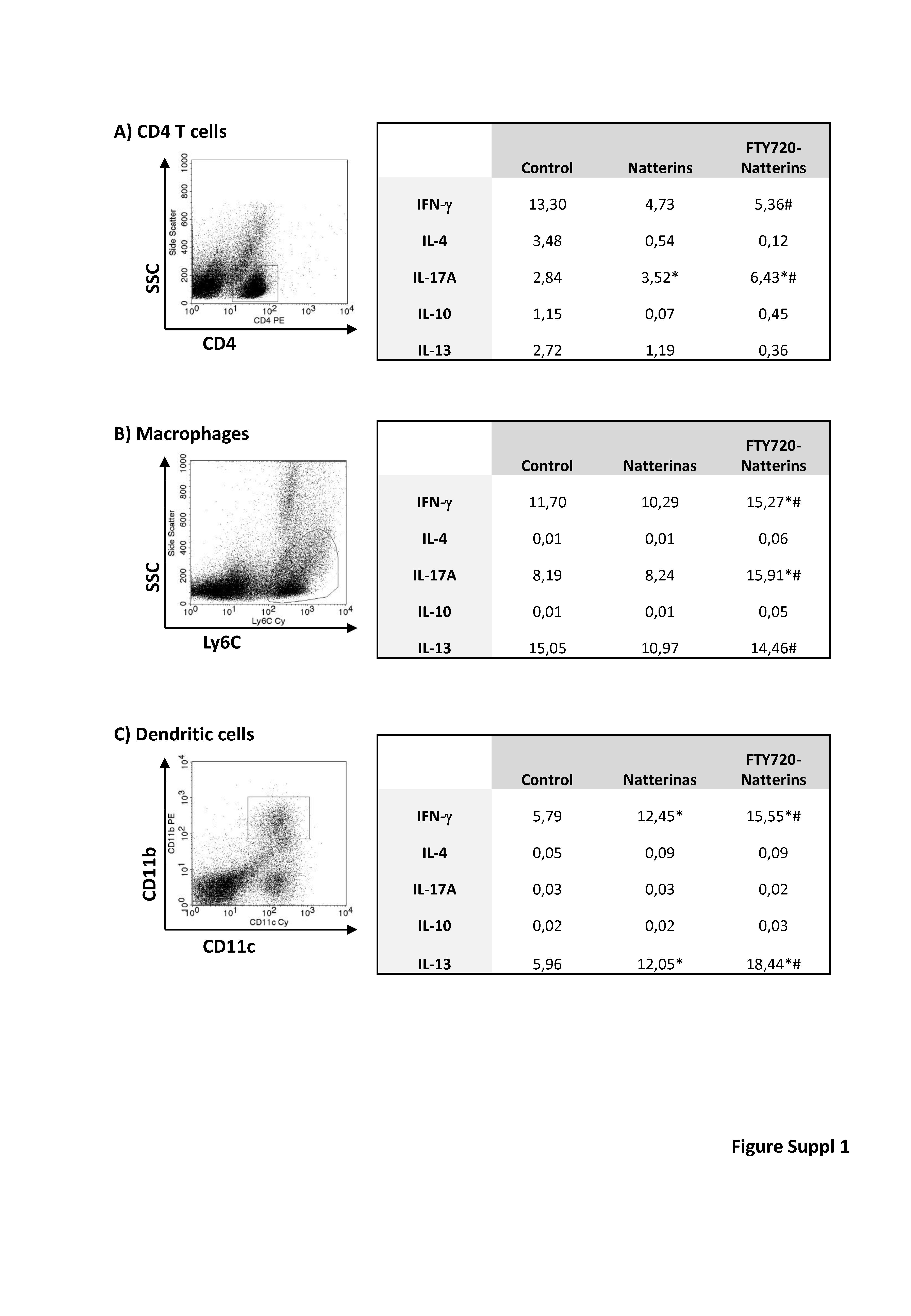

Supplement: Figure S1 — Natterins induce the production of IL-13 and IL-17A by splenic cells. Spleen cells were restimulated for 16 h at 37°C, 5% CO2 with a cell stimulation cocktail containing PMA and ionomycin in the presence of brefeldin A and monesin. Subsequently, after washing and fixation, different subtypes of cells as CD4pos T cells, Ly6Cpos macrophages and CD11bposCD11cpos DC were assessed and for intracellular content of IFN-γ, IL-17A, IL-4, IL-13 and IL-10. The percentages of viable positive-cells that produce cytokine in response to restimulation in culture are shown in tables. *p<0.05 compared to control mice; and # p<0.05 compared to Natterins-immunized mice without FTY720 treatment. (TIFF) [file pone.0071185.s001.tiff]
